# Cytologic Diagnoses of Lung Adenocarcinoma With Concomitant Metastasis From a Different Primary: A Case Series

**DOI:** 10.1002/dc.25460

**Published:** 2025-03-03

**Authors:** Robert Pei, Shane M. Woods, Brant G. Wang

**Affiliations:** ^1^ University of Virginia School of Medicine Inova Campus Falls Church Virginia USA; ^2^ Department of Pathology Inova Fairfax Hospital Falls Church Virginia USA; ^3^ Department of Pathology Georgetown University Medical Center Washington DC USA; ^4^ Department of Pathology and Immunology Baylor College of Medicine Houston Texas USA

**Keywords:** concomitant metastasis, EBUS‐FNA cytology, lung adenocarcinoma, MET exon14 skipping, metastatic ampullary adenocarcinoma, ROS‐1 rearrangement, tumor‐to‐tumor metastasis

## Abstract

Lung adenocarcinoma is a common malignancy that can metastasize. The lung is also a common site for metastasis from other sites. Prompt and accurate diagnosis is critical for patient management. The diagnosis of lung adenocarcinoma can be occasionally challenging due to overlapping clinical and pathological features with adenocarcinomas from other origins. We present three cases of lung adenocarcinomas with concomitant metastatic adenocarcinomas of different primaries in the same endobronchial ultrasound‐guided fine‐needle aspiration (EBUS‐FNA) or core biopsy procedures. The first case showed metastatic ROS‐rearranged lung adenocarcinoma and metastatic ampullary adenocarcinoma involving different mediastinal lymph nodes, respectively, in a patient with no previous history of malignancy. The second case showed metastatic lung adenocarcinoma with MET exon 14 deletion and metastatic breast adenocarcinoma involving different mediastinal lymph nodes, respectively, in a patient with a previous history of breast carcinoma. The third case showed metastatic prostatic adenocarcinoma to a pre‐existing lung mucinous adenocarcinoma in a patient with a previous history of prostatic adenocarcinoma. Our report highlights attention to details, judicious use of immunostains, and ancillary molecular studies in complex pathology cases. Cytohistological findings are also correlated with molecular test results.

## 1 Introduction

Lung adenocarcinoma is a type of non–small‐cell lung carcinoma (NSCLC) and is the most common primary malignancy of the lung, making up about 50% of all lung cancers [1, 2]. It typically manifests as a peripheral lesion with peak incidence between ages 65–75. Typical lung adenocarcinoma presents histologically as a gland‐forming tumor with neoplastic cells often immunoreactive for TTF‐1 [1, 2]. Notably, the lung is also a common site for metastatic malignant tumors; thus, it is essential to differentiate these tumors from primary pulmonary tumors for proper patient management. Here, we report three cases in which the diagnosis of lung adenocarcinoma could be complicated by the presence of a metastatic adenocarcinoma.

### 1.1 Case Report #1

**FIGURE 1:**
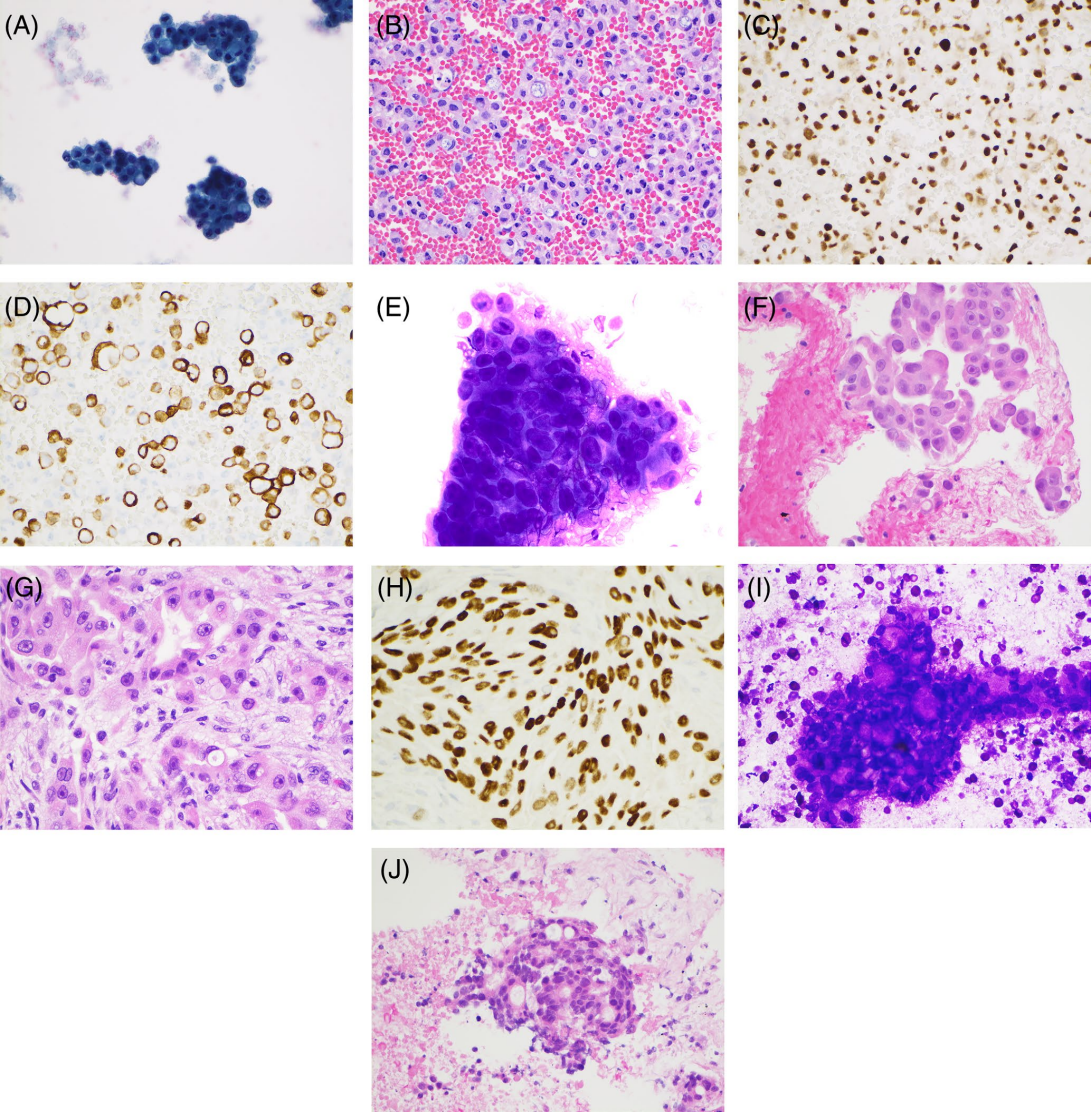
(A) Three‐dimensional clusters of neoplastic cells with intracytoplasmic mucin. Pericardial fluid, pap stain ×400. (B) Clusters of neoplastic cells with intracytoplasmic mucin. Pericardial fluid, cell block H&E stain ×400. (C) Neoplastic cells positive for CDX‐2. Pericardial fluid, immunostain ×400. (D) Neoplastic cells positive for CK20. Pericardial fluid, immunostain ×400. (E) Three‐dimensional clusters of neoplastic cells with prominent nucleoli. EBUS‐FNA of Station 7 lymph node, Diff‐Quik stain ×400. (F) Neoplastic cells with prominent nucleoli. EBUS‐FNA of Station 7 lymph node, cell block H&E stain ×400. (G) Neoplastic cells with prominent nucleoli and abundant vacuolated cytoplasm. Concomitant transbronchial biopsy of right upper lobe mass, H&E stain ×400. (H) Neoplastic cells positive for TTF‐1. Transbronchial biopsy of right upper lobe mass, immunostain ×400. (I) Three‐dimensional clusters of neoplastic cells in a cribriform pattern. EBUS‐FNA of lymph node Station 4L, Diff‐Quik stain ×400. (J) Neoplastic cells in cribriform pattern. EBUS‐FNA of lymph node Station 4L, cell block H&E stain ×400.

A 58‐year‐old female nonsmoker was admitted with obstructive shock secondary to cardiac tamponade and elevated liver enzymes. Pericardiocentesis with Papanicolaou stain revealed clusters of neoplastic cells with intracytoplasmic mucin (Figure 1A) and similar findings on cell block with H&E staining (Figure 1B). Immunostains showed that the neoplastic cells were positive for CDX‐2 (Figure 1C) and CK20 (Figure 1D), and negative for CK7, WT‐1, TTF‐1, PAX‐8, and GATA‐3, supporting the diagnosis of adenocarcinoma with intestinal differentiation. A CT chest study disclosed a 5.5 × 4 cm perihilar mass in the right upper lung lobe with enlarged right hilar and mediastinal lymph nodes measuring up to 3.4 × 2.6 cm with adjacent atelectasis. A CT abdomen with intravenous contrast revealed a periampullary duodenal diverticulum filled with debris, retroperitoneal lymphadenopathy, and ascites. To rule out pericardial metastasis originating from a primary lung neoplasm, the pericardiocentesis cell block was sent to Neogenomics for a lung cancer panel study that revealed no targetable alteration and negative PD‐L1 expression.

Endobronchial ultrasound‐guided fine needle aspiration (EBUS‐FNA) of the Station 7 lymph node demonstrated clusters of neoplastic cells with prominent nucleoli (Figure 1E), with similar findings on the cell block with H&E staining (Figure 1F). Concomitant transbronchial biopsy of the right upper lobe mass revealed an identical finding of prominent nucleoli and vacuolated cytoplasm (Figure 1G). Immunostains showed that the neoplastic cells with prominent nucleoli are positive for TTF‐1 (Figure 1H) and negative for CK20, supporting the diagnosis of lung adenocarcinoma. Similarly, EBUS‐FNA of stations 4L and 4R lymph nodes revealed neoplastic cells with glandular formation (Figure 1I,J), supporting the diagnosis of metastatic adenocarcinoma. NeoGenomics studies on the right upper lobe lung mass biopsy exhibited ROS‐1 rearrangement, PTEN deletion, and high PD‐L1 expression. The patient was treated with immunotherapy (pembrolizumab) and followed by chemotherapy (Carboplatin, pemetrexed, pembrolizumab × 4 cycles) to cover lung adenocarcinoma and a potential gastrointestinal adenocarcinoma. Three‐month follow‐up revealed a reduction in the right lower lobe lung mass and in the mediastinal and right hilar adenopathy.

However, the patient developed jaundice and biliary sepsis. CT abdomen with intravenous contrast showed necrotic retroperitoneal lymphadenopathy, an ampullary mass arising from the aforementioned periampullary duodenal diverticulum filled with debris, and common bile duct dilation to 15 mm. CT‐guided biopsy of the retroperitoneal lymph node and endoscopic ultrasound‐guided fine needle aspiration (EUS‐FNA) of the ampullary mass revealed adenocarcinoma with cytopathological findings resembling those seen in the pericardiocentesis and EBUS‐FNA of mediastinal lymph node stations 4L and 4R, finalizing a diagnosis of metastatic ampullary adenocarcinoma. These findings were differentiated from those seen in EBUS‐FNA of the RUL mass and lymph node Station 7, which were diagnosed as metastatic lung adenocarcinoma with ROS‐1 rearrangement. The patient was additionally treated with abdominal radiation and gemcitabine with pemetrexed, pembrolizumab maintenance therapy. The patient stopped chemotherapy due to a Covid infection and side effects. She passed away 2 years and 5 months after the initial diagnosis.

### 1.2 Case Report #2

An 81‐year‐old female nonsmoker with previously treated, recurrent stage II breast cancer 18 years prior presented with fatigue, dyspnea with exertion, and worsening cough. Laboratory tests were significant for normocytic anemia, hyperglycemia, and hypoalbuminemia with renal dysfunction. CT showed bilateral pleural effusions and a 2.5 × 2.0 cm right lower lobe mass with enlarged mediastinal lymphadenopathy, including a 2.4 × 1.5 cm precarinal lymph node. Multiple brain and bone lesions were also identified in imaging studies.

**FIGURE 2:**
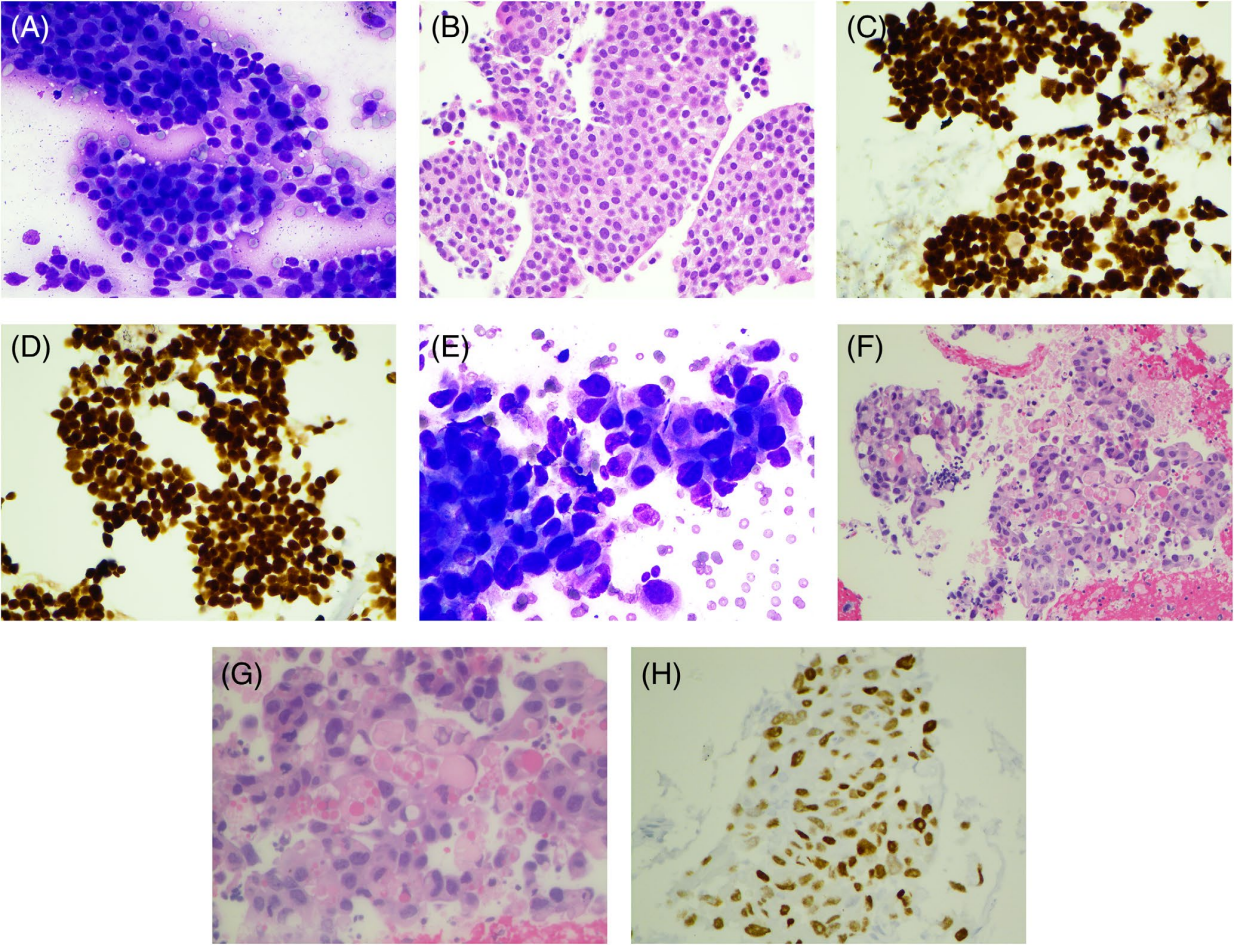
(A) Three‐dimensional clusters of similar‐sized neoplastic cells. EBUS‐FNA of lymph node, Station 4R, Diff‐Quik stain ×400. (B) Similar‐sized neoplastic cells. EBUS‐FNA of lymph node, Station 4R, cell block H&E stain ×400. (C) Neoplastic cells positive for GATA3. EBUS‐FNA of lymph node, Station 4R, immunostain ×400. (D) Neoplastic cells positive for ER. EBUS‐FNA of lymph node, Station 4R, immunostain ×400. (E) Three‐dimensional clusters of neoplastic cells with nuclear pleomorphism and intracytoplasmic vacuoles. EBUS‐FNA of lymph node, Station 7, Diff‐Quik stain ×400. (F) Neoplastic cells with nuclear pleomorphism and intracytoplasmic vacuoles and eosinophilic hyaline globules. EBUS‐FNA of lymph node, Station 7, cell block H&E stain ×200. (G) Neoplastic cells with nuclear pleomorphism and intracytoplasmic eosinophilic hyaline globules. EBUS‐FNA of lymph node, Station 7, H&E stain ×400. (H) Neoplastic cells positive for TTF‐1. EBUS‐FNA of lymph node, Station 7, immunostain ×400.

EBUS‐FNA of the station 4R lymph node revealed clusters of monomorphic neoplastic cells on smear and cell block (Figure 2A,B). Immunostains showed that the neoplastic cells were positive for GATA3 (Figure 2C) and ER (Figure 2D) and negative for synaptophysin, chromogranin, TTF‐1, and PAX8. The findings supported the diagnosis of metastatic breast ductal adenocarcinoma.

In contrast, EBUS‐FNA of the Station 7 lymph node revealed neoplastic cells with nuclear pleomorphism and intracytoplasmic eosinophilic hyaline globules on the cell block (Figure 2E–G). Immunostains showed that the neoplastic cells were positive for TTF‐1 and negative for GATA3 and ER, confirming the diagnosis of concomitant metastatic lung adenocarcinoma (Figure 2H), distinguished from the prior diagnosis of metastatic breast ductal adenocarcinoma. The NGS‐based test on the lung adenocarcinoma from Tempus showed the MET p. D1010H splice region variant Exon 14 deletion‐gain of function (GOF), and the TP53 p. K120E missense variant loss of function (LOF). The patient was on chemotherapy but passed away due to multiple metastases to the liver, bone, abdominal lymph nodes, and brain.

### 1.3 Case Report #3

**FIGURE 3:**
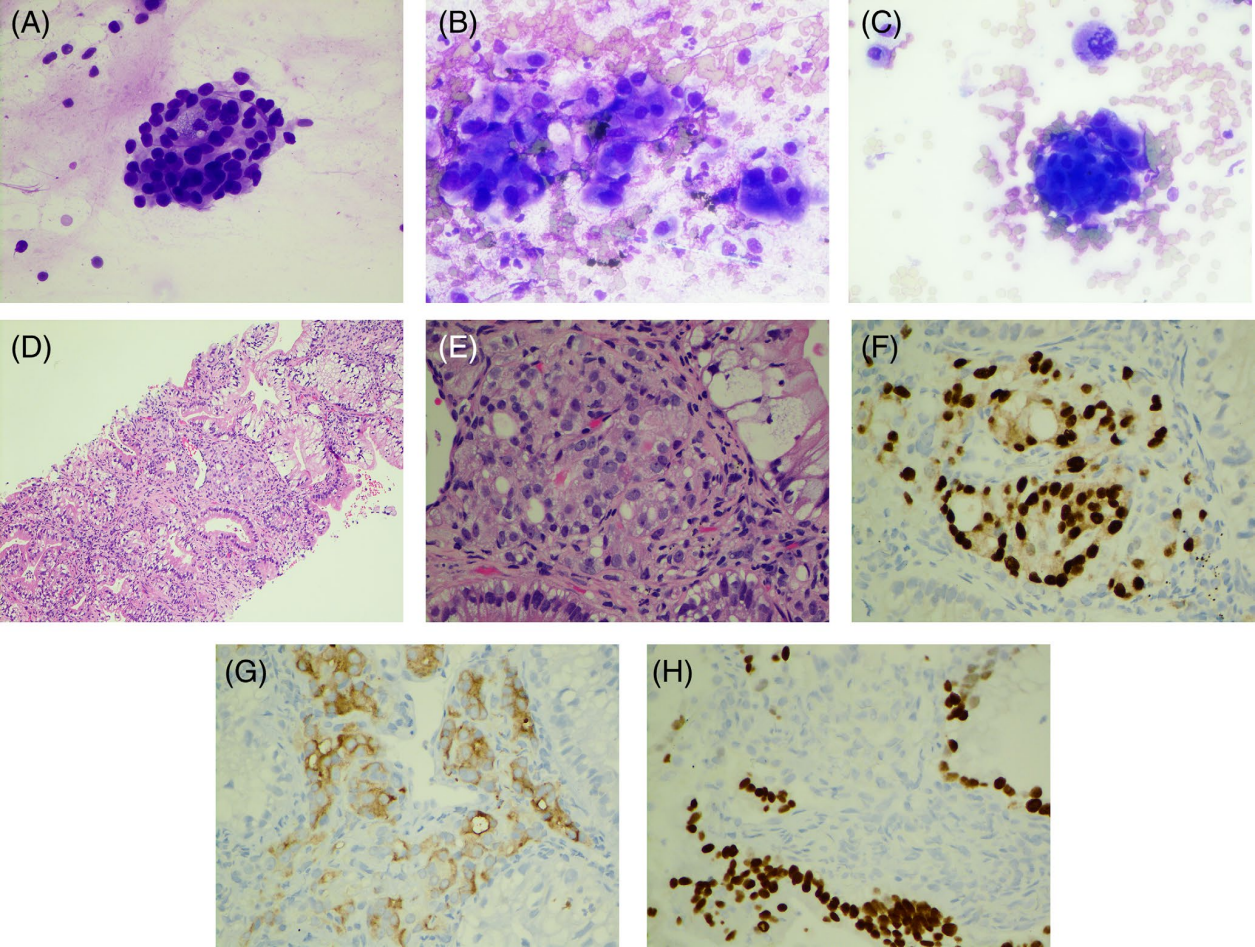
(A) Three‐dimensional cluster of similar‐sized neoplastic cells with acinar formation. EBUS‐FNA of lymph node, Station 7, Diff‐Quik stain ×400. (B) Three‐dimensional clusters of pleomorphic neoplastic cells with abundant cytoplasm. Touch preparation of CT‐guided core needle biopsy of right lower lobe lung nodule, Diff‐Quik stain ×400. (C) Three‐dimensional cluster of similar‐sized neoplastic cells resembling that seen in Figure 3A. Touch preparation of CT‐guided core needle biopsy of the right lower lobe lung nodule, Diff‐Quik stain ×400. (D) Predominantly mucinous adenocarcinoma with a focal area of acinar formation. CT‐guided core needle biopsy of the right lower lobe lung nodule, H&E stain ×100. (E) High power view of neoplastic cells with acinar formation and prominent nucleoli. CT‐guided core needle biopsy of the right lower lobe lung nodule, H&E stain ×400. (F) Neoplastic cells positive for NKX3.1, CT‐guided core needle biopsy of right lower lobe lung nodule, immunostain ×400. (G) Neoplastic cells positive for PSA. CT‐guided core needle biopsy of right lower lobe lung nodule, immunostain ×400. (H) Neoplastic cells positive for CDX‐2 in the background of mucinous adenocarcinoma, CT‐guided core needle biopsy of the right lower lobe lung nodule, immunostain ×400.

A 71‐year‐old male nonsmoker with a past medical history of metastatic prostatic adenocarcinoma to the cervical and thoracic spine presented for clinical follow‐up. His original prostatectomy 13 years prior demonstrated prostatic adenocarcinoma Gleason score 3 + 4 = 7 with negative margins. Initial chest CT showed extensive right lower lobe (RLL) pulmonary infiltrate suspicious for pneumonia. No solid lung metastatic disease was identified at this time. Follow‐up chest CT 3 months later revealed persistent airspace disease in the RLL, denser than on prior examination with the densest part measuring 10.8 cm surrounded by ground‐glass opacities, as well as a 1.9 cm nodule in the right upper lobe (RUL) adjacent to the major fissure. EBUS‐FNA of the Station 7 lymph node showed monomorphic neoplastic cells in an acinar formation (Figure 3A). Immunostains revealed that the neoplastic cells were positive for NKX‐3.1 and negative for TTF1, confirming the diagnosis of metastatic prostatic adenocarcinoma.

At the same encounter, EBUS‐FNA of the RLL lung mass showed adenocarcinoma with mucinous features, morphologically different from that seen in the Station 7 lymph node as shown in Figure 3A. In addition, immunostains showed that the neoplastic cells in the RLL mass were positive for CK7 and CDX2 and negative for NKX‐3.1 and TTF1.

In order to obtain a more representative sample, CT‐guided biopsy of the RLL mass was performed. Touch preparation of CT‐guided core needle biopsy with Diff‐Quik staining of the RLL mass showed a large number of neoplastic cells with abundant cytoplasm and identical morphology as that seen in EBUS FNA of the RLL mass (Figure 3B). Interestingly, minimal neoplastic cells with a similar morphology to those seen in Figure 3A were also noted (Figure 3C). On H&E staining, the core biopsy showed neoplastic cells with prominent nucleoli and acinar formation (Figure 3D) within a background of mucinous adenocarcinoma composed of neoplastic cells with abundant mucinous cytoplasm (Figure 3E).

Immunostains showed that some of the neoplastic cells in Figure 3D were positive for NKX3.1 (Figure 3F), PSA (Figure 3G), and negative for CK7 and CDX‐2, confirming the presence of metastatic prostatic adenocarcinoma. In contrast, immunostains showed that the neoplastic cells of the mucinous adenocarcinoma were positive for CK7, Napsin‐A (focal), and CDX‐2 (Figure 3H), negative for NKX3.1 and PSA. These findings support the diagnosis of tumor‐to‐tumor metastasis (prostatic adenocarcinoma into lung mucinous adenocarcinoma). Furthermore, NGS‐based test from Tempus revealed TMPRSS2‐ERG chromosomal rearrangement that can be typically seen in prostatic adenocarcinoma, in addition to findings more commonly seen in lung adenocarcinoma, such as RBM10 c.725‐1G>C splice region variant loss of function (LOF), CDKN2A copy number loss, and MTAP copy number loss. The patient underwent chemotherapy and radiation therapy and was alive 6 months after initial EBUS‐FNA diagnosis.

## 2 Discussion

Lung adenocarcinoma has well‐defined cytopathological findings due to its prevalence. When combined with clinical findings, this tends to lead to a reliable diagnosis [1, 2]. However, the presence of metastatic adenocarcinoma from a different primary site creates pathological findings that can make diagnosis challenging and delay treatment pathways. This case series scrutinized situations where additional concomitant metastatic adenocarcinomas were present, with or without the patient's prior history of adenocarcinoma, and where two different tumors occupied the same space, all of which could potentially prolong the clinical workup leading to the final diagnosis and proper management.

Cytohistological differences can be appreciated with careful examination in all three cases described in this report. In case #1, the metastatic ampullary adenocarcinoma showed monomorphic neoplastic cells arranged in a cribriform pattern and an immunophenotype typical of adenocarcinoma of intestinal differentiation (CK20 and CDX‐2 positive and CK7 negative). In contrast, the lung adenocarcinoma showed neoplastic cells with abundant vacuolated/eosinophilic cytoplasm, prominent nucleoli, and TTF‐1 immunoreactivity, findings that could be seen in ROS‐1 rearranged lung adenocarcinoma [3]. In case #2, metastatic breast ductal adenocarcinoma showed relatively monomorphic cells immunoreactive for GATA3 and ER. In contrast, the metastatic lung adenocarcinoma showed neoplastic cells with marked nuclear pleomorphism, intracytoplasmic eosinophilic hyaline globules, and TTF‐1 immunoreactivity, findings typically seen in lung adenocarcinoma with MET exon 14 skipping in an elderly female nonsmoker [4]. In case #3, despite the diagnosis of metastatic prostatic adenocarcinoma on EBUS‐FNA of the Station 7 lymph node, we struggled to make sense of the EBUS‐FNA findings of the right lower lobe lung mass showing adenocarcinoma with mucinous features, noting that prostatic adenocarcinoma could also have mucinous features [5]. Later, a touch preparation of the core biopsy of the right lower lobe lung mass yielded similar clusters of monomorphic neoplastic cells resembling those seen in metastatic prostatic adenocarcinoma identified in the Station 7 lymph node. Careful examination of the core biopsy demonstrated clusters of metastatic prostatic adenocarcinoma within a preexisting lung mucinous adenocarcinoma, confirmed by respective immunostain profiles. The neoplastic cells of metastatic prostatic adenocarcinoma were positive for NKX3.1 and PSA, negative for CDX‐2, CK7, and TTF1; whereas the neoplastic cells of lung mucinous adenocarcinoma were positive for CDX2, CK7, and napsin‐A (focal), negative for TTF1, NKX3.1, and PSA. This was further confirmed by NGS‐based test results [6, 7, 8, 9, 10].

One may argue that the presentation of adenocarcinomas of different morphology and immunostain profile is due to intratumoral heterogeneity instead of bona fide different primaries in cases #1 and #3. In case #1, compared to ampullary intestinal‐type adenocarcinoma, lung enteric adenocarcinomas are more likely to be CK7 and TTF1 immunoreactive, and they are more likely to be seen in older male smokers [11, 12]. In addition, ROS1‐rearranged lung adenocarcinomas are more common in younger female nonsmokers [3]. The presentation of ampullary mass, retroperitoneal lymphadenopathy, and ascites favors a diagnosis of primary ampullary adenocarcinoma. In case #3, the mucinous component of the lung mass was unlikely to be mucinous prostatic adenocarcinoma since mucinous prostatic adenocarcinoma with this morphology has low Gleason grades and is thus unlikely to metastasize. Furthermore, mucinous prostatic adenocarcinoma tends to have a better prognosis than conventional prostatic adenocarcinoma [5]. NGS test results of the mucinous component were in line with lung mucinous adenocarcinoma [8, 9, 10]. Although CDKN 2A copy number loss and MTAP copy number loss are frequently associated with mesothelioma [13], either MTAP copy number loss or CDKN 2A copy number loss can be seen in lung adenocarcinoma independently [10, 14].

We describe three cases in which EBUS‐FNA of the mediastinal lymph nodes and lung masses revealed adenocarcinoma of different origins and one case with metastatic prostatic adenocarcinoma to a preexisting lung mucinous adenocarcinoma. Although metastasis of one adenocarcinoma to multiple sites is more likely, one needs to pay attention to cytohistological details and apply immunohistochemical studies judiciously to reach correct diagnoses, especially if there is clinically rapid tumor growth and progression. Molecular tests of the tumors may also confirm the specific diagnoses and provide opportunities for targeted therapy [15, 16].

## Data Availability

The data that support the findings of this study are available on request from the corresponding author. The data are not publicly available due to privacy or ethical restrictions.
